# Development and Characterization of Phage Display-Derived Monoclonal Antibodies to the S2 Domain of Spike Proteins of Wild-Type SARS-CoV-2 and Multiple Variants

**DOI:** 10.3390/v15010174

**Published:** 2023-01-06

**Authors:** Ji Woong Kim, Ah Hyun Cho, Ha Gyeong Shin, Sung Hoon Jang, Su Yeon Cho, Ye Rim Lee, Sukmook Lee

**Affiliations:** 1 Department of Applied Chemistry, Kookmin University, Seoul 02707, Republic of Korea; 2 Biopharmaceutical Chemistry Major, School of Applied Chemistry, Kookmin University, Seoul 02707, Republic of Korea; 3 Antibody Research Institute, Kookmin University, Seoul 02707, Republic of Korea

**Keywords:** SARS-CoV-2, spike protein, phage display, sandwich immunoassay

## Abstract

The rapid emergence of new severe acute respiratory syndrome coronavirus 2 (SARS-CoV-2) variants has resulted in the ongoing global coronavirus disease 2019 (COVID-19) pandemic. Thus, the rapid development of a platform to detect a broad range of SARS-CoV-2 variants is essential for successful COVID-19 management. In this study, four SARS-CoV-2 spike protein-specific single-chain variable fragments (scFvs) were isolated from a synthetic antibody library using phage display technology. Following the conversion of these scFvs into monoclonal antibodies (mAbs) (K104.1–K104.4) and production and purification of the mAbs, the antibody pair (K104.1 and K104.2) that exhibited the highest binding affinity (K104.1 and K104.2, 1.3 nM and 1.9 nM) was selected. Biochemical analyses revealed that this antibody pair specifically bound to different sites on the S2 subunit of the spike protein. Furthermore, we developed a highly sensitive sandwich immunoassay using this antibody pair that accurately and quantitatively detected the spike proteins of wild-type SARS-CoV-2 and multiple variants, including Alpha, Beta, Gamma, Delta, Kappa, and Omicron, in the picomolar range. Conclusively, the novel phage display-derived mAbs we have developed may be useful for the rapid and efficient detection of the fast-evolving SARS-CoV-2.

## 1. Introduction

The ongoing coronavirus disease 2019 (COVID-19) pandemic is caused by severe acute respiratory syndrome coronavirus 2 (SARS-CoV-2), a novel coronavirus [1]. Ever since the initial SARS-CoV-2 outbreak in early December 2019, high-frequency mutations in SARS-CoV-2 have altered the infectivity of the virus and led to the emergence of a variety of SARS-CoV-2 variants, including Alpha, Beta, Gamma, Delta, Kappa, and Omicron [2,3]. As of January 2023, approximately 662 million confirmed COVID-19 cases and more than 6.7 million deaths have been reported worldwide [4]. Early detection of SARS-CoV-2 spread is vital for the prevention and management of COVID-19 [5]. Hence, the development of novel, accurate, and sensitive SARS-CoV-2 detection methods is essential for the prevention of viral transmission and timely management of COVID-19.

Currently, three basic types of tests are used to detect SARS-CoV-2 infection: tests based on (i) viral RNA detection, (ii) viral antigen detection, and (iii) detection of antibodies against the virus [6,7]. Viral RNA detection is conducted using nucleic acid amplification tests (NAATs), such as reverse transcription-polymerase chain reaction (RT-PCR), which is considered to be the reference standard for the diagnosis of SARS-CoV-2 infection [8,9]. Although NAATs have excellent sensitivity and provide confirmatory COVID-19 diagnosis, it requires trained personnel, expensive equipment, sensitive reagents, and a relatively long procedure [10,11]. SARS-CoV-2 point-of-care antigen tests based on sandwich immunoassay, a highly sensitive and specific viral antigen detection method, are also used as rapid mass screening tools to detect SARS-CoV-2 infection [12,13,14]. In contrast to NAATs, these tests are relatively inexpensive and less time-consuming, with fewer chances of specimen manipulation [15]. In addition, other point-of-care diagnostic approaches have also been developed for the rapid detection of SARS-CoV-2 viral antigen [16,17,18,19]. However, the rapid emergence of SARS-CoV-2 variants has hampered the utility of SARS-CoV-2 point-of-care antigen tests [20]. Therefore, development of a novel method for the detection of a broad range of SARS-CoV-2 variants is imperative to effectively cope with the emergence and re-emergence of SARS-CoV-2 variants.

The SARS-CoV-2 spike protein is composed of two main subunits, N-terminal S1 and C-terminal S2, with a furin cleavage site (S1/S2 cleavage region) [21]. The S1 subunit contains a receptor-binding domain (RBD) that is critical for its interaction with the human angiotensin-converting enzyme II, a well-known host cell receptor for SARS-CoV-2 infection [22]. The S2 subunit plays a key role in mediating viral cell fusion and integration into the host cells [23]. Recently, many antibodies against the S1 subunit have been developed. However, multiple variants of SARS-CoV-2 with a high number of mutations have been reported to be closely associated with antibody escape [24]. Furthermore, recent studies revealed that compared to the S1 subunit, the S2 subunit is more conserved [25]. Therefore, the development of monoclonal antibodies (mAbs) specifically targeting the S2 subunit may provide a better solution to detect a broad range of SARS-CoV-2 variants.

In this study, for the rapid development of mAbs for the detection of the constantly changing SARS-CoV-2, we isolated SARS-CoV-2 spike protein-specific single-chain variable fragment (scFv) antibody clones from an established antibody library using phage display technology. Following conversion of these scFvs into mAbs and subsequent biochemical characterization of the mAbs, the antibody pair (comprising of a capture antibody and detection antibody) that exhibited the highest affinity to the different binding sites of the S2 subunit was selected. Furthermore, through the development of a sandwich immunoassay using this antibody pair, the accuracy, sensitivity, and utility of the novel immunoassay for the detection of wild-type SARS-CoV-2 and multiple variants were evaluated. The novel mAbs developed in this study may be useful platforms for the rapid detection of a broad range of SARS-CoV-2 variants.

## 2. Materials and Methods

### 2.1. Selection of scFvs Using Phage Display Technology

*Escherichia coli* strain ER2738 cells harboring phagemids encoding the scFv genes from a previously constructed synthetic scFv library [26] were cultured in 1.6 L of super broth (SB) supplemented with 50 μg/mL of ampicillin and incubated at 37 °C with constant agitation until the OD600 of 0.6 was achieved. Then, 1 × 10^13^ plaque-forming units (pfu) of VCSM13 helper phage (Agilent, Santa Clara, CA, USA) was added and incubated at 37 °C for 2 h. Subsequently, 70 μg/mL of kanamycin was added into the media and incubated at 37 °C overnight with constant agitation. Then, the rescued phages were precipitated from the supernatant by adding 4% (*w*/*v*) polyethylene glycol-8000 and 3% (*w*/*v*) NaCl. After centrifugation for 40 min at 12,000× *g* at 4 °C, the phages were resuspended in 3% (*w*/*v*) bovine serum albumin (BSA) in phosphate-buffered saline (PBS). Using the rescued scFv-displayed phage pool, biopanning was performed to select SARS-CoV-2 extracellular domain of S protein (SARS-CoV-2 S-ECD)-specific scFvs from a synthetic scFv antibody library, as previously described [27]. Briefly, four rounds of biopanning were conducted to select SARS-CoV-2 S-ECD-specific scFv clones using magnetic beads (Dynabeads M-270 Epoxy; Invitrogen, Waltham, MA, USA) coated with 4 μg recombinant SARS-CoV-2 S-ECD (Sino#40589-V08B1; Sino Biological Inc., Beijing, China). Ninety-six clones were randomly selected from colonies on the output plate, and their reactivity to SARS-CoV-2 S-ECD was evaluated using phage enzyme-linked immunosorbent assay (ELISA).

### 2.2. Phage ELISA

Individual colonies of the ninety-six clones selected from the fourth round of biopanning were inoculated into 1 mL of SB containing 50 μg/mL of carbenicillin in 96-deep-well plates (Axygen, Union City, CA, USA) and incubated at 37 °C for 5 h. Subsequently, 10^10^ pfu of VCSM13 phages (Agilent) were added to the plates and incubated overnight at 37 °C. The plates were centrifuged at 2000× *g*, and the phage supernatant was used for ELISA. Briefly, 96-well high binding microplates (Corning, Corning, NY, USA) were coated with 0.1 μg of SARS-CoV-2 S-ECD (Sino#40589-V08B1) or 3% BSA in PBS and incubated overnight at 4 °C. After blocking using 3% (*w*/*v*) BSA in PBS, the plates were incubated with 100 μL of phage supernatant at 37 °C for 2 h. After three rounds of washing with PBS containing 0.05% (*v*/*v*) Tween 20 (PBST), horseradish peroxidase (HRP)-conjugated anti-hemagglutinin (HA) antibody (1:3000; Bethyl Laboratories, Montgomery, TX, USA) was added and incubated at 37 °C for 1 h. Colorimetric detection was performed using 3,3′,5,5′-tetramethylbenzidine (TMB) substrate solution (Thermo Fisher Scientific, Waltham, MA, USA). The reaction was terminated by the addition of 1 M H_2_SO_4_, and absorbance was measured at 450 nm using a microtiter plate reader (Bio-Tek Instruments, Winooski, VT, USA).

### 2.3. DNA Cloning

The selected scFv clones were converted to IgG-based mAbs using DNA cloning. Each of the heavy and light chain variable region genes of the scFv clones was individually amplified using PCR with the following forward and reverse primers for heavy chains: 5′-AGTGTGCTGGAATTCGCTGCCACTATGGAATGGAGCTGGGTCTTTCTCT TCTTCCTGTCAGTAACAACAGGTGTCCTTTCCGAGGGCAGCTGTTGGAGTCTGGG G-3′ and 5′-AAGACCGATGGGCCCTTGGTTGAGGCTGAGCTCACGGTGACCAGTGTACCC-3′, respectively; and forward and reverse primers for light chains, 5′-TGCA GCCACCGTACGTAGGACCGTCAGCTTGGTGCCTCCG-3′ and 5′-GGGAGACCCAA GCTTGCCGCAACCATGGAGACACATTCCCAGGTCTTTGTATATATGTTGCTGTGG CTTTCAGGCGTTGAAGGGCAGTCTGTGCTGACTCAGCCACCCT-3′, respectively. Each of the heavy and light chain PCR fragments was cloned into a pcDNA3.1 mammalian expression vector (Thermo Fisher Scientific) that encoded human IgG1.

To generate HA-tagged IgG-based mAbs, the HA-tag sequence (YPYDVPDYA) was incorporated into the C-terminus of the fragment crystallizable region of human IgG1. Each of the heavy and light chain variable region genes of the scFv clones was subcloned into the HA-tagged vector as described above.

### 2.4. Cell Culture

Expi293F cells were purchased from Thermo Fisher Scientific. The cells were cultured in Expi293™ expression medium (Gibco, Waltham, MA, USA) and maintained in a shaking incubator in an 8% CO_2_ atmosphere at 37 °C with constant agitation.

### 2.5. Preparation of SARS-CoV-2 Spike Protein-Specific IgG-Based mAbs

To produce SARS-CoV-2 spike protein-specific IgG-based mAbs, recombinant vectors encoding the selected IgG-based mAbs were transfected into Expi293F cells using the ExpiFectamine 293 transfection kit (Thermo Fisher Scientific) following the manufacturer’s instructions. Briefly, the ExpiFectamine 293 transfection reagent and pcDNA3.1 vector encoding the selected antibodies were individually incubated in Opti-MEM (Gibco) for 5 min. Then, the transfection reagent and vectors were combined, further incubated for 20 min, and transfected into the cells. Seven days after transfection, the resulting supernatants were collected for antibody purification using affinity chromatography with protein A Sepharose column (Cytiva, Marlborough, MA, USA), as previously described [28]. Briefly, 1 mL of pre-packed protein A Sepharose column was equilibrated with PBS. Then, the antibody supernatant was loaded onto the column at a flow of 0.5 mL/min. After washing with PBS with 20-column volume, the antibodies were eluted using 0.1 M glycine-HCl at pH 3.0. Following dialysis in PBS, the size and purity of antibodies were confirmed using sodium dodecyl-sulfate polyacrylamide gel electrophoresis (SDS-PAGE) and Coomassie brilliant blue (CBB) staining. The purity of antibodies was quantified using the Image J software (National Institutes of Health, MD, USA).

### 2.6. Surface Plasmon Resonance (SPR)

Real-time measurement of the binding kinetics between SARS-CoV-2 spike protein and the selected mAbs was analyzed using an iMSPR mini-instrument (icluebio, Seongnam, South Korea). The SARS-CoV-2 S-ECD was immobilized on a research-grade carboxyl (COOH) sensor chip (icluebio) using an amine coupling kit (icluebio) according to the manufacturer’s instructions. Increasing concentrations (8, 16, 32, 64, and 128 nM) of the selected mAbs in running buffer containing 10 mM (2-(4-(2-hydroxyethyl)piperazin-1-yl)ethanesulfonic acid)-buffered saline (pH = 7.4), 2 mM CaCl_2_, 1 mM MnCl_2_, 700 mM NaCl, and 0.005% (*v*/*v*) Tween 20 were then injected at a flow rate of 50 µL/min at 25 °C. The sensor chips were regenerated during each cycle by injecting 10 mM glycine-HCl (pH = 2.5) to remove surface-bound mAbs. The association constant (K_a_), dissociation constant (K_d_), and equilibrium constant (K_D_; K_D_ = K_d_/K_a_) were calculated using the iMSPR analysis software (icluebio).

To confirm the additional binding of the detection antibody (K104.2-HA) to SARS-CoV-2 S-ECD after binding of the capture antibody (K104.1) to the S-ECD reached saturation, 512 nM of K104.1 and K104.2-HA were sequentially exposed to the immobilized SARS-CoV-2 S-ECD at a flow rate of 50 µL/min at 25 °C.

### 2.7. ELISA

Each well of a ninety-six well high binding microplate was coated with 0.1 μg of recombinant SARS-CoV-2 S1 (Sino#40591-V08B1), S2 (Sino#40590-V08B), and S-ECD (Sino#40589-V08B1) proteins, followed by blocking with 3% (*w*/*v*) BSA in PBS for 2 h, and then incubated with 15 μg/mL of mAbs for 2 h at 25 °C. The plates were then washed three times with PBST, and antigen-bound mAbs were detected using HRP-conjugated anti-Fc antibody (1:5000, Thermo Fisher Scientific). After three rounds of washing with PBST, TMB substrate solution was added to each well and reaction with HRP was allowed to take place for 5 min. The reaction was terminated by the addition of 100 μL of 1 M H_2_SO_4_. Absorbance was measured at 450 nm using a microplate reader (Bio-Tek Instruments).

### 2.8. Competition ELISA

SARS-CoV-2 S-ECD (Sino#40589-V08B1) (0.1 μg) was coated onto a ninety-six well high binding microplate and incubated overnight at 4 °C. After several rounds of washing with PBST, the plate was blocked using 3% (*w*/*v*) BSA in PBS for 2 h and pre-incubated in the absence or presence of increasing amounts (0.021, 0.062, 0.18, 0.56, 1.67, 5, or 15 μg) of the isotype control (Invitrogen#31154) or the selected antibodies (K104.1, K104.2, K104.3, or K104.4) for 1 h at 25 °C. The plates were then washed with PBST and incubated with 0.15 μg of HRP-conjugated K104.1 for 1 h at 25 °C. After several rounds of washing with PBST, the TMB substrate solution was added to each well and reaction with HRP was allowed to take place for 5 min. The reaction was terminated by the addition of 100 μL of 1 M H_2_SO_4_. Absorbance was measured at 450 nm using a microplate reader (Bio-Tek Instruments).

### 2.9. Sandwich Immunoassay

Ninety-six-well high binding microplates coated with the capture antibody (K104.1) were blocked using 3% (*w*/*v*) BSA in PBS for 2 h at 37 °C. Next, 100 μL of increasing concentrations of the S-ECD of wild-type MERS-CoV (Sino#40069-V08B), SARS-CoV (Sino#40634-V08B), SARS-CoV-2 (Sino#40589-V08B1), or SARS-CoV-2 variants [Alpha (Sino#40589-V08B6), Beta (Sino#40589-V08B7), Gamma (Sino#40589-V08B10), Delta (Sino# 40589-V08B16), Kappa (Sino#40589-V08B15), and Omicron (Sino#40589-V08B33)] were added to each well and incubated for 3 h at 37 °C. The plates were washed three times with PBST and incubated with HA-tagged detection antibody (K104.2-HA) for 1 h at 37 °C. After three rounds of washing with PBST, the HA-tag was detected using an HRP-conjugated anti-HA antibody (1:3000, Bethyl Laboratories). After three rounds of washing with PBST, the TMB substrate solution was added to each well and reaction with HRP was allowed to take place for 5 min. The reaction was terminated by the addition of 100 μL of 1 M H_2_SO_4_. Absorbance was measured at 450 nm using a microplate reader (Bio-Tek Instruments).

### 2.10. Statistical Validation of the Sandwich Immunoassay

Intra-assay precision was determined by measuring sample triplicates run six times in the same assay run. The inter-assay precision was determined by measuring a sample in triplicate in six separate assay runs. The mean and standard deviation (SD) values were calculated to determine the coefficient of variation (CV), which was calculated as follows: CV (%) = (SD/mean) × 100. The percentage recovery of ELISA was calculated as follows: (average measured concentration/expected concentration) × 100.

## 3. Results

### 3.1. Selection and Biochemical Characterization of SARS-CoV-2 Spike Protein-Specific IgG-Based mAbs

**Figure 1:**
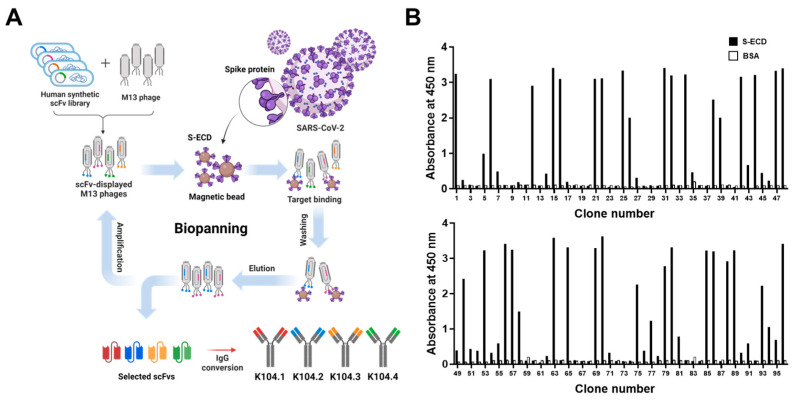
Isolation of severe acute respiratory syndrome coronavirus-2 (SARS-CoV-2) spike protein-specific IgG-based monoclonal antibodies (mAbs). (**A**) Schematic representation of the selection and preparation of SARS-CoV-2 spike protein-specific mAbs. Biopanning based on phage display technology using immobilized magnetic beads of SARS-CoV-2 extracellular domain of S protein (S-ECD) was used to select four SARS-CoV-2 spike protein-specific single-chain variable fragments (scFvs) from a synthetic scFv library, which were then converted to IgG-based mAbs (K104.1–K104.4). (**B**) The SARS-CoV-2 S-ECD-specific binding ability of ninety-six clones randomly selected after the four rounds of biopanning was determined using phage enzyme-linked immunosorbent assay (ELISA). Bovine serum albumin (BSA) was used as the negative control.

Biopanning was first performed using phage display technology to select novel SARS-CoV-2 spike protein-specific antibodies from a synthetic scFv antibody library. Based on DNA sequencing, four scFv clones possessing complementarity-determining regions with different amino acid sequences were selected. Following the conversion of the selected scFvs into IgG-based mAbs, IgG-based mAbs (K104.1–K104.4) were prepared (Figure 1A). Phage ELISA revealed that the selected scFv clones strongly bound to SARS-CoV-2 S-ECD but did not bind to BSA (negative control), indicating the SARS-CoV-2 spike protein specificity of the selected scFvs (Figure 1B). After production and purification, SDS-PAGE and CBB staining were performed to determine the purity of the IgG-based mAbs. The purity was found to be more than 90% (data not shown).

**Figure 2:**
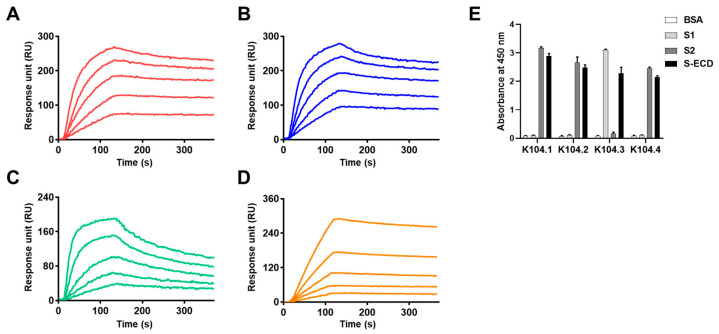
Biochemical analyses of the selected IgG-based mAbs. The binding affinities of the selected mAbs to SARS-CoV-2 S-ECD were analyzed using surface plasmon resonance (SPR) measurements. Each SPR sensorgram shows the interaction between different concentrations (8 nM, 16 nM, 32 nM, 64 nM, and 128 nM) of (**A**) K104.1, (**B**) K104.2, (**C**) K104.3, and (**D**) K104.4 and the SARS-CoV-2 S-ECD-immobilized sensor chip. (**E**) BSA (white bars), S1 (light gray bars) and S2 (dark grey bars) subunits, and S-ECD (black bars) were coated onto microtiter microplates. ELISA was used to measure the molecular specificity of the selected mAbs.

Real-time kinetic analyses based on SPR measurements were carried out to determine the binding affinity of the selected IgG-based mAbs to the SARS-CoV-2 spike protein. The K_D_ for K104.1, K104.2, K104.3, and K104.4 based on the SPR results were found to be 1.28 nM, 1.90 nM, 8.22 nM, and 12.21 nM, respectively (Figure 2A–D and Table 1), indicating the high binding affinity (in the nanomolar range) of the selected IgG-based mAbs to the SARS-CoV-2 spike protein. Furthermore, to confirm the molecular specificity of the selected IgG-based mAbs, each of the mAbs (K104.1–K104.4) was added to different wells of S1-, S2-, and S-ECD-coated microplates, and their reactivity was measured using ELISA. The results showed that K104.1, K104.2, and K104.4 were specifically bound to the S2 subunit, whereas K104.3 was bound to the S1 subunit (Figure 2E).

### 3.2. Selection of an Antibody Pair Comprising of Antibodies with Different Epitopes and High Binding Affinity to SARS-CoV-2 Spike Protein

**Figure 3:**
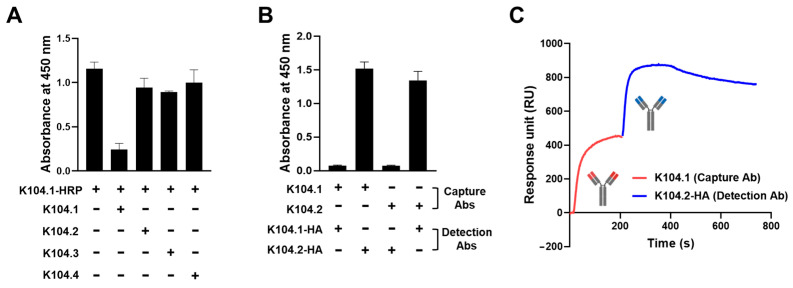
Identification of a suitable SARS-CoV-2 spike protein-specific antibody pair for sandwich immunoassay. (**A**) Competition ELISA to identify mAbs that do not compete with K104.1 for binding to SARS-CoV-2 S-ECD. Results are represented as mean ± SD of duplicates and represent one out of two independent experiments. (**B**) Sandwich immunoassay conducted using the untagged forms of K104.1 or K104.2 as capture antibodies and anti-hemagglutinin (HA)-tagged K104.1 (K104.1-HA) and K104.2 (K104.2-HA) as detection antibodies. Results are represented as mean ± standard deviation (SD) of duplicates from one out of two independent experiments. (**C**) Additional binding of K104.2-HA (blue) to SARS-CoV-2 S-ECD saturated with K104.1 (red) was analyzed using SPR measurements. This is a representative curve of two independent experiments.

To develop a sandwich immunoassay, a set of two antibodies, each possessing non-overlapping epitopes and distinct binding sites on the same antigen of interest, are required [29]. Thus, we performed a competition ELISA to identify which IgG-based mAb (K104.2, K104.3, and K104.4) has binding sites on SARS-CoV-2 S-ECD that differ from K104.1, the antibody with the highest binding affinity to S-ECD. Following the pre-incubation of 15 μg of the selected mAbs with SARS-CoV-2 S-ECD, prepared HRP-conjugated K104.1 was then incubated. None of the three IgG-based mAbs (K104.2, K104.3, and K104.4) that bound to S-ECD competed with HRP-conjugated K104.1, indicating that binding sites of these antibodies are different from that of K104.1 (Figure 3A). To further confirm the specificity of this competition, we also performed competition ELISA by increasing the concentrations of isotype control or selected mAbs and obtained similar results to those shown in Figure 3A (Supplementary Figure S1).

To determine whether K104.1 and K104.2, the two antibodies that exhibited the highest binding affinity to SARS-CoV-2 S-ECD and possessed different binding sites on SARS-CoV-2 S-ECD, are a suitable antibody pair for sandwich immunoassay, we generated and prepared HA-tagged K104.1 or K104.2 (K104.1-HA and K104.2-HA). Untagged forms of K104.1 and K104.2 were used as capture antibodies and immobilized onto a microtiter plate, followed by incubation with S-ECD. The reactivity was measured in the presence and absence of K104.1-HA or K104.2-HA as detection antibodies. The results showed that both pairs (K104.1-K104.2-HA and K104.2-K104.1-HA) are suitable antibody pairs for sandwich immunoassays (Figure 3B). Additionally, to analyze the independent binding of the selected mAb pair to SARS-CoV-2 S-ECD, we performed competition assays using SPR measurements. The results showed that binding of K104.2-HA to SARS-CoV-2 S-ECD-immobilized sensor chip was observed even after K104.1 saturation on the chip, indicating that K104.1 and K104.2-HA in the selected antibody pair have different binding sites on the SARS-CoV-2 spike protein (Figure 3C).

### 3.3. Development and Characterization of Sandwich Immunoassay for the Detection of Wild-Type SARS-CoV-2 Spike Protein

**Figure 4:**
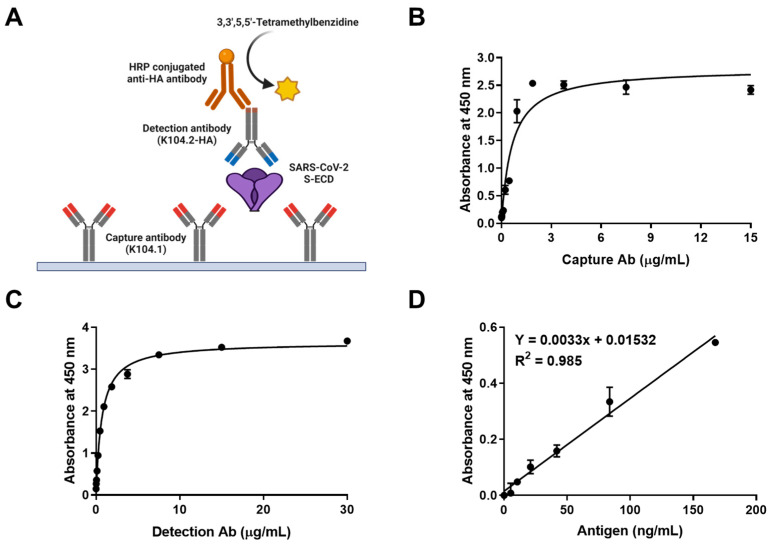
Development and characterization of SARS-CoV-2 spike protein-specific sandwich immunoassay. (**A**) Schematic representation of the sandwich immunoassay. (**B**) Optimization of the capture antibody (K104.1) concentration using the sandwich immunoassay. Microplate wells were coated with varying concentrations of K104.1, followed by incubation of the plates with 0.1 μg of SARS-CoV-2 S-ECD and 5 μg/mL of detection antibody (K104.2-HA). (**C**) 3.75 μg/mL of K104.1 was coated onto a microplate and incubated with 0.1 μg of SARS-CoV-2 S-ECD. The optimum concentration of the detection antibody (K104.2-HA) was determined using sandwich immunoassay titration with varying concentrations of K104.2-HA. (**D**) SARS-CoV-2 S-ECD calibration curve to determine the limit of detection (LOD) was constructed using 1.875 μg/mL of K104.1 and 3.75 μg/mL of K104.2-HA. All values represent mean ± SD of triplicate measurements from one out of the six independent experiments.

Sandwich immunoassay is a reliable tool for the rapid detection and mass screening of infectious viral antigens [30,31]. A SARS-CoV-2 S-ECD-specific sandwich immunoassay was developed using K104.1 and K104.2-HA as the capture and detection antibody, respectively. Specific recognition of the selected mAb pair by the SARS-CoV-2 S-ECD was analyzed using chemiluminescent ELISA (Figure 4A).

Optimization of capture and detection antibody concentrations is a key factor in determining the sensitivity and working range of sandwich immunoassays [32]. The reactivity of various concentrations of the capture and detection antibodies was evaluated in the microplate-based sandwich immunoassay to determine the optimum concentrations. The results showed that the optimum concentration for the capture (K104.1) and detection (K104.2-HA) mAbs were 1.875 μg/mL and 3.75 μg/mL, respectively (Figure 4B,C).

The linear dynamic range of the calibration curve with the SARS-CoV-2 S-ECD was found to be between 0 ng/mL and 167.5 ng/mL (equivalent to 0 nM–1.2 nM). The reproducibility of the calibration curve was demonstrated using six independent assays. The limit of detection (LOD) of SARS-CoV-2 S-ECD was 43.2 ng/mL (equivalent to 320 pM) (Figure 4D). The intra- and inter-assay CVs and recovery values of the optimized sandwich immunoassay were calculated. The intra- and inter-assay CVs for 50 ng/mL of SARS-CoV-2 S-ECD were 7.17% and 7.28%, respectively. The intra- and inter-assay recovery values for 50 ng/mL SARS-CoV-2 S-ECD were calculated to be 102.25 and 97.95%, respectively (Table 2). Intra- and inter-assay variations of less than 10% were acceptable, suggesting that the sandwich immunoassay developed in this study may be a sensitive, accurate, and reliable technique for quantitatively measuring SARS-CoV-2 spike protein [33].

### 3.4. Detection of the Spike Proteins of Multiple SARS-CoV-2 Variants and Other Coronaviruses

**Figure 5:**
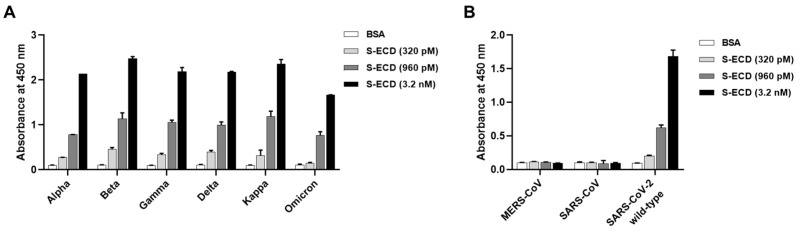
Detection of the spike proteins of wild-type MERS-CoV, SARS-CoV, and SARS-CoV-2 and variants using the developed sandwich immunoassay. Detection efficiency of the established sandwich immunoassay was evaluated in the absence (using negative control BSA) and presence of increasing concentrations of the spike proteins of (**A**) SARS-CoV-2 variants (Alpha, Beta, Gamma, Delta, Kappa, and Omicron) or (**B**) wild-type MERS-CoV, SARS-CoV, and SARS-CoV-2. All values represent mean ± SD of triplicate measurements from one out of two independent experiments.

To evaluate the utility of the developed sandwich immunoassay against multiple SARS-CoV-2 variants, the assay was performed in the presence and absence of increasing concentrations (320 pM [LOD for SARS-CoV-2 wild-type S-ECD], 960 pM, and 3.2 nM) of SARS-CoV-2 variants, including Alpha (B.1.1.7), Beta (B.1.351), Gamma (P.1), Delta (B.1.617.2), Kappa (B.1.617.1), and Omicron (BA.1). The results showed that the developed sandwich immunoassay was highly sensitive (in the picomolar range) to the spike proteins of all six tested SARS-CoV-2 variants (Figure 5A). To further confirm the specificity of the assay, we performed the sandwich immunoassay using S-ECDs of wild-type MERS-CoV, SARS-CoV, and SARS-CoV-2. Results showed that the immunoassay only detects the S-ECD of SARS-CoV-2 but not those of MERS-CoV and SARS-CoV, further indicating the specificity of the developed sandwich immunoassay for SARS-CoV-2 variants (Figure 5B).

## 4. Discussion

Ever since the initial SARS-CoV-2 outbreak in 2019, the rapid emergence and continuous spread of SARS-CoV-2 variants harboring novel mutations have resulted in the ongoing pandemic with unprecedented global healthcare and socioeconomic implications [34,35]. Thus, a novel platform for the early detection and mass screening of individuals infected with SARS-CoV-2 variants is essential for the effective management of COVID-19 and prevention of viral transmission [36]. In the present study, we selected a novel SARS-CoV-2 S2 subunit-specific antibody pair (K104.1 and K104.2) comprising antibodies possessing different epitopes using phage display technology-based antibody selection. In detail, K104.1 and K104.2 were selected as capture and detection antibodies, respectively, because the SPR analysis showed that K104.1 has a higher affinity to SARS-CoV-2 S-ECD than K104.2. In addition, our sandwich immunoassay results also revealed that K104.1 (capture antibody)-K104.2 (detection antibody) pair was slightly more sensitive than the K104.2 (capture antibody)-K104.1 (detection antibody) pair. Thus, we developed a sensitive sandwich immunoassay using this antibody pair that accurately detected the spike proteins of multiple SARS-CoV-2 variants. This study validated the effectiveness of the novel sandwich immunoassay as a feasible platform for the detection of multiple SARS-CoV-2 variants.

Recent advances in recombinant DNA technology have opened new avenues for the rapid development of mAbs [37,38]. Conventional antibodies are generated using a combination of immunization and hybridoma technology, which is time-consuming and labor-intensive [39]. In this study, we demonstrated how phage display technology-based antibody selection from an established antibody library can be an effective alternative for the rapid development of reliable and accurate antibodies for the early detection of novel viruses. Our findings from this study support this hypothesis. First, IgG-based mAbs (K104.1 and K104.2) selected from a synthetic antibody library (size ~7 × 10^9^) exhibited a binding affinity (based on SPR analyses) of approximately 1 nM to SARS-CoV-2 S-ECD. Second, the highly specific sandwich immunoassay developed using this antibody pair (K104.1 as the capture antibody and K104.2-HA as the detection antibody, each binding to different sites on the S-ECD) could accurately detect the spike proteins of multiple SARS-CoV-2 variants. The LOD of the sandwich immunoassay for SARS-CoV-2 wild-type S-ECD was in the picomolar range. The intra- and inter-assay CVs further validated the accuracy and reliability of the sandwich immunoassay. Furthermore, most previously developed sandwich immunoassays detect viral antigens of SARS-CoV-2 in nano- or picomolar ranges, which is comparable to the results of our immunoassay [40,41,42]. For example, Svobodova et al. [43] and Dominico et al. [44] reported the LOD values of the sandwich immunoassays as ~270 pM and ~1 nM, respectively. Thus, the results from this study provide insights into the development of novel antigen detection platforms for the rapid and accurate detection of SARS-CoV-2 infection.

To date, numerous antibodies targeting the S1 subunit of SARS-CoV-2 have been developed; however, most of these antibodies are unreactive to SARS-CoV-2 variants [45,46]. In contrast, the selected antibodies in this study specifically bound to the S2 subunit, but not the S1 subunit. Furthermore, the developed sandwich immunoassay that utilizes an S2 subunit-specific antibody pair was found to be highly sensitive to the S-ECDs of multiple SARS-CoV-2 variants, including Alpha, Beta, Gamma, Delta, Kappa, and Omicron. Therefore, these observations led us to theorize that the S2 subunit may be a promising target for the development of mAbs for the detection of multiple SARS-CoV-2 variants. Our hypothesis was validated by earlier studies. First, Kudriavtsev et al. [47] reported that the highest mutation rate was observed in the RBD sequence of the S1 subunit in SARS-CoV-2 variants. Second, 80% of the total mutations occur in the S1 subunit of prevalent SARS-CoV-2 variants, including Alpha, Beta, Gamma, Delta, and Kappa, and the current Omicron variant, whereas less than 20% of mutations occur in the S2 subunit (Supplementary Figure S2) [48,49]. Our findings collectively indicate the effectiveness of the sandwich immunoassay using novel IgG-based mAbs developed in this study as a useful platform for the detection of multiple SARS-CoV-2 variants.

## 5. Conclusions

In conclusion, this study described phage display-based scFv selection, selection of an IgG-based mAb pair for sandwich immunoassay, and the development of a novel highly sensitive sandwich immunoassay that accurately detects the spike protein of wild-type SARS-CoV-2. Furthermore, we also demonstrated the utility of the developed sandwich immunoassay for the detection of novel and resurgent SARS-CoV-2 variants such as Alpha, Beta, Gamma, Delta, Kappa, and Omicron. To the best of our knowledge, this is the first report on the development of a sandwich immunoassay for the detection of spike proteins of wild-type SARS-CoV-2 and multiple variants. However, the current study has not used clinical samples for the validation of the developed sandwich immunoassays. In the near future, we plan to determine the specific epitopes of the selected IgG-based mAbs, validate the specificity and sensitivity of this technology using clinical samples, and further optimize the assay as a SARS-CoV-2 platform for the detection of pan-SARS-CoV-2 variants.

## Figures and Tables

**Table 1:** Evaluation of the binding kinetics of the selected IgG-based mAb to SARS-CoV-2 S-ECD.

mAb	^1^K_D_ (nM)	^2^K_a_ (1/M^−1^s^−1^)	^3^K_d_ (s^−1^)
K104.1	1.28	3.18 × 10^5^	4.08 × 10^−4^
K104.2	1.90	4.04 × 10^5^	7.68 × 10^−4^
K104.3	8.22	2.87 × 10^5^	2.36 × 10^−3^
K104.4	12.21	3.15 × 10^4^	3.85 × 10^−4^

^1^K_D_, equilibrium dissociation constant; ^2^K_a_, association constant; ^3^K_d_, dissociation constant.

**Table 2:** Reliability of the developed sandwich immunoassay for the detection of SARS-CoV-2 S-ECD.

Spiked Level (ng/mL)	Intra-Assay (*n* = 6)			Inter-Assay (*n* = 6)		
	Mean ± SD (ng/mL)	Recovery (%)	CV (%)	Mean ± SD (ng/mL)	Recovery (%)	CV (%)
50	51.12 ± 3.58	102.25	7.17	48.97 ± 3.57	97.95	7.28

SD, standard deviation; CV, coefficient of variation.

## Data Availability

The datasets used and/or analyzed during the current study are available from the corresponding author on reasonable request.

## Supplementary Materials

The following supporting information can be downloaded at: https://www.mdpi.com/article/10.3390/v15010174/s1, Figure S1: Identification of a non-competing antibody pair against SARS-CoV-2 spike protein. Competition ELISA was conducted by pre-incubating SARS-CoV-2 S-ECD with increasing concentrations of isotype control or the selected mAbs, followed by incubating with prepared HRP-conjugated K104.1. All values represented as mean ± SD of triplicate measurements and represent one out of two independent experiments. Figure S2: The schematic diagram of the spike protein of SARS-CoV-2 wild-type and multiple variants with mutations. Black lines indicate the mutation sites on each S1 and S2 subunit of SARS-CoV-2 spike proteins. Each mutation rate of S1 or S2 subunit within the spike proteins of SARS-CoV-2 variants are indicated, respectively. NTD: N-terminal domain, RBD: receptor-binding domain, FP: fusion peptide, HR1: heptapeptide repeat sequence 1, HR2: heptapeptide repeat sequence 2.
