# Supplementary material for: Development and Characterization of Phage Display-Derived Monoclonal Antibodies to the S2 Domain of Spike Proteins of Wild-Type SARS-CoV-2 and Multiple Variants
Source: Viruses. 2023 Jan 6;15(1):174. doi: 10.3390/v15010174 (PMC9862430; doi:10.3390/v15010174)
Supplement: Supplementary file 1 [file viruses-15-00174-s001.zip › viruses-2027624-supplementary.pdf]

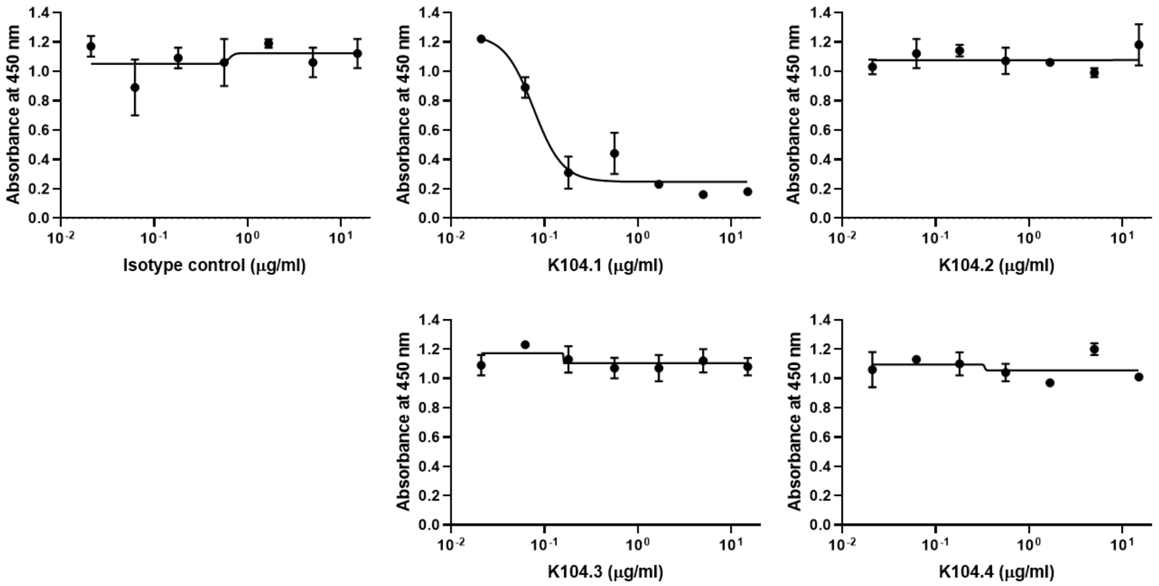

Supplementary Figure S1. Identification of a non-competing antibody pair against SARS-CoV-2 spike protein. Competition ELISA was conducted by pre-incubating SARS-CoV-2 S-ECD with increasing concentrations of isotype control or the selected mAbs, followed by incubating with prepared HRP-conjugated K104.1. All values represented as mean ± SD of triplicate measurements and represent one out of two independent experiments.

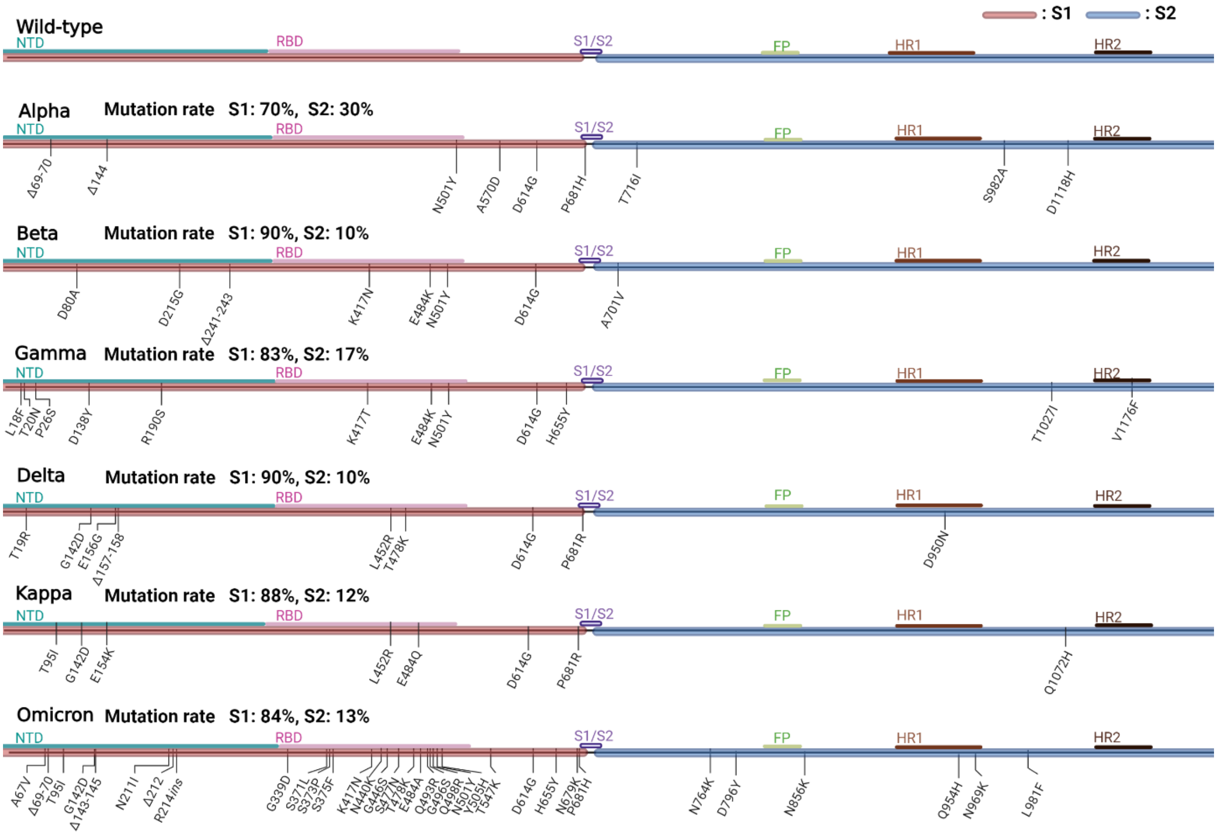

Supplementary Figure S2. The schematic diagram of the spike protein of SARS-CoV-2 wild-type and multiple variants with mutations. Black lines indicate the mutation sites on each S1 and S2 subunit of SARS-CoV-2 spike proteins. Each mutation rate of S1 or S2 subunit within the spike CoV-2 variants are indicated, respectively. NTD: N-terminal domain, RBD: receptor-binding domain, FP: fusion peptide, HR1: heptapeptide repeat sequence 1, HR2: heptapeptide repeat sequence 2.
